# Validity of the German Version of the Continuous-Scale Physical Functional Performance 10 Test 

**DOI:** 10.1155/2017/9575214

**Published:** 2017-07-09

**Authors:** Irene Härdi, Stephanie A. Bridenbaugh, M. Elaine Cress, Reto W. Kressig

**Affiliations:** ^1^University Center for Medicine of Aging and Rehabilitation, Felix Platter-Hospital, Basel Mobility Center, Basel, Switzerland; ^2^Department of Kinesiology, Institute of Gerontology, University of Georgia, Athens, GA, USA

## Abstract

**Background:**

The Continuous-Scale Physical Functional Performance 10 Test (CS-PFP 10) quantitatively assesses physical functional performance in older adults who have a broad range of physical functional ability. This study assessed the validity and reliability of the CS-PFP 10 German version.

**Methods:**

Forward-translations and backtranslations as well as cultural adaptions of the test were conducted. Participants were German-speaking Swiss community-dwelling adults aged 64 and older. Concurrent validity was assessed using Pearson correlation coefficients between CS-PFP 10 and gait velocity, Timed Up and Go Test, hand grip strength, SF-36 physical function domain, and Freiburger Physical Activity Questionnaire. Internal consistency was calculated by Cronbach's alpha.

**Results:**

Backtranslation and cultural adaptions were accepted by the CS-PFP 10 developer. CS-PFP 10 total score and subscores (upper body strength, upper body flexibility, lower body strength, balance and coordination, and endurance) correlated significantly with all measures of physical function tested. Internal consistency was high (Cronbach's alpha 0.95–0.98).

**Conclusion:**

The CS-PFP 10 German version is valid and reliable for measuring physical functional performance in German-speaking Swiss community-dwelling older adults. Quantifying physical function is essential for clinical practice and research and provides meaningful insight into physical functional performance of older adults. This trial is registered with ClinicalTrials.gov NCT01539200.

## 1. Introduction

The ability to perform activities of daily living such as walking, dressing, or carrying objects is crucial for functional independence. Physical functional limitations in older adults are often strong predictors for disability, nursing home admission, and death [1]. Maintenance of functional independence is a primary goal of older adults, their care providers, and the health-care system. The first step in preventing functional decline is to determine current physical functional performance and beginning deficits [2]. A variety of functional measures exists, from self-reported questionnaires to performance-based assessments of selected tasks [3]. Instruments such as the “Short Physical Performance Battery” [4] or the “Physical Performance Test” [5] measure mobility and balance in older adults but they only provide a partial view of functional performance with little insight into the extent to which the individual is actually restricted in performing everyday activities. Many physical performance measures have floor or ceiling effects [6] which restrict their applicability to a certain range of physical functional performance. Tests of physical functional performance applicable to frail populations often lack meaningful outcomes when used in healthy, vigorous older adults because of an instrument's ceiling effects. Although clinical experience in the evaluation of function is valuable and important, subjective measures of performance can bias scoring and interpretation as well as limit the comparability of results. The quantification of physical function is a crucial addition to clinical practice and clinical research, particularly in providing objective measures and measures of changes over time. It is greatly beneficial to be able to use the same assessment tool in people with a broad range of physical functional ability so that it is applicable for frail, vulnerable, and vigorous populations.

The Continuous-Scale Physical Functional Performance Test (CS-PFP) was developed in the United States by Professor Elaine Cress (MEC) to accurately, precisely, and objectively assess functional performance in older adults across a broad spectrum of abilities [7]. The English version of the CS-PFP and its short version, the Continuous-Scale Physical Functional Performance 10 Test (CS-PFP 10), are standardized, validated, reliable, and objective measures of physical function in everyday tasks in ambulatory older adults [8]. The CS-PFP 10 addresses a wide range of activities that are important for independence in older adults using 10 common tasks such as picking up something from the floor, transferring laundry from the washer to the dryer, and climbing stairs [7, 8]. From the perspective of evidence-based research, it is essential to have the instrument of interest available in the native language of those being tested and that this instrument undergoes the process of validation to become a widely accepted tool for assessing physical function in older adults with higher physical ability. Furthermore, in order to allow cross-cultural comparisons of study outcomes, the instruments must not only be well-translated, linguistically speaking, but also adapted culturally to maintain the content validity of the instrument [9].

The aims of this study were to describe the translation process as well as the site set-up of the CS-PFP 10 laboratory in a German-speaking region of Switzerland. Furthermore, the study aimed to investigate the concurrent validity and the internal reliability of the German version of the CS-PFP 10 in a sample of community-dwelling, self-reported healthy, older adults.

## 2. Methods

### 2.1. Study Design and Population

The present study (validation of the German version of the CS-PFP 10) was part of a randomized placebo-controlled parallel group trial. The main study investigated the effects of 6-month once daily oral whey-protein supplementation in combination with a once weekly Jaques-Dalcroze Eurhythmics training (multicomponent music-based group exercise) on functionality, probability of independence, and/or functional reserve among community-dwelling seniors compared to the effects of 6-month Jaques-Dalcroze Eurhythmics training alone (study abbreviation “NUDAL,” ClinicalTrials.gov identifier NCT01539200). Community-dwelling older adults were recruited through information and recruitment events in regional senior centers as well as advertisements in local newspapers and in the local radio of the Canton Basel City. The training sessions took place at different senior centers throughout the Canton Basel City. The baseline data was collected between February 2012 and May 2013. The baseline data used for this substudy was extracted from the entire database of the main study. The study—the main study as well as the present substudy—was approved by the local ethics committee. Written informed consent was given by each participant before study participation.

Inclusion criteria were as follows: (I) community-dwelling males and females, (II) age ≥ 64 years, (III) being able to walk unassisted for 15 meters, and (IV) Mini Mental State Examination (MMSE) score ≥ 24 points. Exclusion criteria were as follows: (I) serum 25(OH) vitamin D > 100 nmol/L from the blood sampling at baseline, (II) current regular (defined as >3x/week) use of high protein oral nutritional supplements, (III) milk protein allergy, (IV) lactose intolerance, (V) severe visual impairment (corrected near vision < 0.2 in both eyes), (VI) severe neurological, orthopedic, rheumatologic, or psychiatric illness causing inability to understand or follow task instructions or to walk 15 meters without assistance, and (VII) terminal illness with life expectancy less than 12 months, as determined by a physician.

### 2.2. Procedures

All tests were performed at the Basel Mobility Center or at the Clinical Trial Unit at the University Hospital Basel by trained research assistants or the study physician. Baseline testing consisted of two test batteries which occurred on two separate days maximum 14 days apart. The first test battery included the informed consent, Mini Mental State Examination, the Freiburger Physical Activity Questionnaire, and 36-item Short-Form General Health Survey (SF-36) version 1. The second test battery included the demographic and health data, physical examination, and the functional measurements—hand grip strength, Timed Up and Go Test, gait analysis, and CS-PFP 10.

### 2.3. Development of the German CS-PFP 10

The translation and cross-cultural adaptation of the original English version of the CS-PFP 10 into German were performed in two respects. First, the test instructions were translated from English to German. Second, the CS-PFP 10 site set-up was culturally adapted, using typically Swiss household items and products (see Site Set-Up).

#### 2.3.1. Test Instructions

The translation of the CS-PFP 10 from English to German was done by one medical doctor (native language American English, fluent German speaker) who was familiar with the objectives of the test. The backtranslation from German to English was done by a different researcher (native language American English, fluent German speaker) who had no prior knowledge of the instrument, was blind to the original version of the CS-PFP 10 instructions, and yet was familiar with health-related topics. MEC, the developer of the CS-PFP 10, examined the backtranslation and approved the test instructions.

#### 2.3.2. Site Set-Up

The site set-up was done in accordance with the “Specification of Measurement by Task” document available on the CS-PFP website (http://www.drelainecress.com/). The CS-PFP 10 consists of 10 everyday tasks and, therefore, requires diverse equipment. To fulfill the requirements of the set-up guidelines, most of the equipment was purchased in the USA, for example, Posey® safety belts, pylons, cooking pots (task 1), jackets (task 2; white hip-length lab jackets are used at our site), and tote bags (task 9), or was constructed just for our laboratory, for example, stair platform (task 8 and 9) that fulfills the stair tread height and depth requirements. However, with approval by MEC, some of the household appliances, accessories, and products were typical of the local culture. For the floor sweep (task 5), a horsehair broom and a hand brush with a short-handled dustpan was used instead of a broom with synthetic fibers and a long handled dustpan. For the first part of sixth task, the laundry loading task, a top-loading washing machine was replaced by a front-loading washing machine. The grocery items (task 9) were purchased in the Swiss chain grocery stores Coop and/or Migros so that the food and household products were easily recognized by our study participants. These accepted changes to the site set-up protocol made the devices and food products recognizable for the older adults being tested, which made the simulated tasks (such as grocery shopping or sweeping the floor) more realistic. One exception, approved by MEC, was that the sand bags (task 1) were filled with lead granulates; however, the weight and the number of sandbags remained in accordance with the site set-up guidelines.

### 2.4. Functional Measurements and Questionnaires

#### 2.4.1. CS-PFP 10

The Continuous-Scale Physical Functional Performance 10 Test was developed to quantify information about physical functionality of older adults performing everyday tasks. The test consists of 10 tasks which are carried out under standard conditions and in a predetermined order of increasing intensity and difficulty (see Table 1). A detailed description of the tasks has been reported elsewhere [7]. For each task, the objective values of “time,” “weight,” or “distance” are measured. The participants are asked to perform each task safely and comfortably but as quickly as possible and with maximal effort. The execution of the task itself is determined by the participants' judgment. The participants are guided by a test administrator throughout the whole test. The instructions, the timing, and other measurements of the tasks and the data entry are standardized. To ensure safety, a Posey safety belt was placed around each patient's waist for easy grasp by the test administrator to prevent a fall.

The measured results are manually entered in the assessment tool software. The software converts the raw data into a scaled score between zero and 100 and provides a total score of the CS-PFP 10, as well as five subscores—upper body strength (UBS), lower body strength (LBS), upper body flexibility (UBF), balance and coordination (B&C), and endurance (END). The total score is the average of all variables. A higher score indicates a higher level of functioning. The CS-PFP value of 57 points or higher is predictive of a physical reserve and independent living status [10]. The original CS-PFP 10 (English version) is a valid, reliable, and sensitive measure with no floor or ceiling effects [7, 8, 11].

#### 2.4.2. Gait Assessment

The spatiotemporal parameters of gait were collected with the GAITRite® system (GAITRite Platinum, CIR System, Sparta, NJ, USA, Version 4.7), a 10-meter-long electronic walkway with integrated pressure sensors [12]. The walking trials were performed according to the European guidelines for spatial-temporal gait analysis [13]. The gait analysis for the NUDAL study consisted of six different walking tasks, whereas walking at habitual, self-selected walking speed (referred to as normal walking in this text) represented the first and second task. Results of both normal walking speed tasks were averaged to be used for analysis. Details regarding the description of the gait analysis at the Basel Mobility Center have been reported elsewhere [14]. Before testing, a trained evaluator gave standardized verbal instructions regarding the test procedure. In order to measure steady-state gait, the patients initiated and terminated each walk 1.25 m before and after the 10 m walkway allowing sufficient distance to accelerate and decelerate. No practice trials were performed. To ensure safety, a Posey safety belt was placed around each patient's waist for easy grasp by the trained evaluator who walked behind the patients during all trials. The patients performed all trials wearing their own footwear. A video camera was used during the gait analysis to allow detailed review. General conditions of the described gait assessment were also reported in a previous article of the principal author [15]. Gait velocity (cm/s) during normal walking (average of two trials) was the parameter of interest for this substudy.

#### 2.4.3. Timed Up and Go Test

Basic mobility was assessed with the Timed Up and Go Test (TUG) [16]. The TUG measures the time in seconds that it takes an individual to rise from a chair with armrests, walk three meters at the person's habitual, self-selected speed, turn, walk back, and sit down again. Older adults who require 14 seconds or longer to complete the task have a high risk for falls [17].

#### 2.4.4. Hand Grip Strength

Hand grip strength was measured in the dominant hand with a GRIP-A T.K.K. 5001 Grip Strength Dynamometer®. The participants performed the test while sitting comfortably with shoulder adducted and neutrally rotated, the elbow flexed to 90 degrees, forearm and wrist in neutral position [18]. The participants were instructed to perform a maximal isometric contraction. The test was repeated after 10 seconds and the higher value (kg) was recorded for data analysis [19]. Reference values for hand grip strength are commonly presented according to age, gender, and side specificity. The average normal value for females aged 70–74 years has been reported to be 24.2 kg for the right-hand side [20].

#### 2.4.5. Freiburger Physical Activity Questionnaire

This self-report questionnaire assesses levels of basic physical activity (e.g., gardening, climbing stairs), leisure time physical activity (e.g., dancing, bowling), and sports activity (e.g., jogging, swimming) in the previous week or in the previous month. Significant test-retest reliability was reported for the summed physical activity level (*r* = 0.56), in people between the ages of 18 and 78 years. Cross-correlation with maximum oxygen uptake revealed a significant correlation coefficient of *r* = 0.42 [21]. For the present study the short version of the questionnaire was used [22] and the parameter of interest was the overall activity (total time in hours per week).

#### 2.4.6. SF-36

Self-perceived physical function was assessed using the validated 36-item Short-Form Health Survey (SF-36) version 1 [23]. The questionnaire consists of 36 questions, is self-administered, and assesses quality of life and well-being in eight multi-item scales regarding physical functioning and perception of physical role, vitality, general and mental health, perception of emotional role, social functioning, and bodily pain. For the present study only the physical function domain (SF-36 PF), which consists of 10 items, was assessed. Each item is scaled between 0 and 100 with higher scores reflecting higher self-perceived function.

### 2.5. Clinical Evaluation and Assessments

Clinical assessment included the demographic and health data as well as a physical examination. The following data was used for the present study: age, sex, height, weight, falls (defined as unintentionally coming to rest on the ground or other surfaces [24]) in the preceding 12 months (single-item question), fear of falling (single-item question), and relevant medical problems.

### 2.6. Statistical Analysis

Characteristics of baseline assessment, functional measurements, and questionnaires were summarized descriptively using either means and standard deviations or frequencies and percentages, as appropriate.

The concurrent validation, a subtype of criterion-related validity, of the German version of the CS-PFP 10 was assessed by examining correlation between the CS-PFP 10 (total score and its subscores) and the gait velocity, TUG, hand grip strength, Freiburger Physical Activity Questionnaire, and the SF-36 physical function domain. Correlation was performed using the Pearson correlation coefficient. A correlation value greater than 0.50 indicates a large relationship, a value between 0.50 and 0.30 indicates a moderate relationship, and a value between 0.30 and 0.1 indicates a small relationship [25]. Probability values less than 0.05 were considered statistically significant.

Internal reliability of the German version of the CS-PFP 10 was evaluated by calculating Cronbach's alpha coefficient [26] for each subscore and the total score. Ceiling and floor effects of the CS-PFP 10 and its subscores were determined by reaching the minimum or maximum score.

Analyses were conducted using the SPSS version 22.0 (IBM Corp., Armonk, NY) software program for Windows.

## 3. Results

### 3.1. Development of the German Version of the CS-PFP 10

The translation and backtranslation of the instructions proceeded without difficulties. All participants understood the test and task instructions. For the CS-PFP 10 site set-up, all equipment fulfilled the prescribed guidelines. Either they were purchased in the USA to have the same style and sizes used in the original CS-PFP laboratory or culturally specific Swiss items were used, as approved by MEC and described in the Methods.

The developer of the original CS-PFP 10 version (MEC) visited our CS-PFP 10 site at the Basel Mobility Center in Basel and approved the backtranslation of the test instructions, the set-up of the CS-PFP 10 laboratory, and the cultural adaptions. No alterations were necessary. The Basel Mobility Center is a certified CS-PFP 10 site. Stephanie A. Bridenbaugh (coauthor) is a certified CS-PFP test administrator and a CS-PFP test trainer.

### 3.2. Descriptive Characteristics of Study Population and Measurements

Table 2 provides the characteristics of the 109 participants included in this substudy. The mean age of the community-dwelling participants was 74.1 ± 6.4 years with an age range of 64–89, and 91 (83.5%) were women.

On average, the participants completed the CS-PFP 10 in approximately 50 minutes. No participants fell or were injured during testing. The mean CS-PFP 10 total score was 44.1 ± 16.2 points, ranging from 9.1 to 84.7 points (Table 3). The performance of task seven “sit down and get up from the floor” was declined by 6 participants (5.5%). Reasons stated were problems with herniated disc (*n* = 1) and concerns about their ability to kneel (*n* = 5; osteoarthritis in knees (*n* = 1), subjective inability after knee or hip surgery (*n* = 4)). For those who declined or were unable to perform this function, a score of zero was entered for this task. All other tasks were carried out by the participants. In total, 87 participants (79.8%) had a total score below the threshold score of 57 points, indicating low physical function for carrying out daily activities independently.

No ceiling or floor effects were observed because no participants obtained the highest (100 points) or lowest (0 points) score.

### 3.3. Validity and Reliability of the German CS-PFP 10

#### 3.3.1. Concurrent Validity

**Figure 1:**
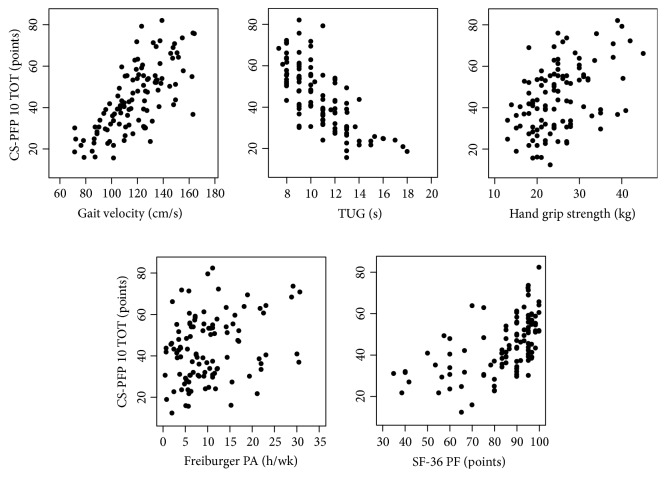
Scatter plots of the CS-PFP 10 total score and the concurrent validity measures.

Pearson's product moment correlation coefficients between the CS-PFP 10 total score and subscores and gait velocity, TUG time, hand grip strength, Freiburger Physical Activity Questionnaire results, and SF-36 PF scores are provided in Table 4. The CS-PFP 10 total score as well as the subscores correlated significantly with each of the concurrent validation measures. Scatter plots of the correlations are presented in Figure 1. The total scores' correlations with gait velocity, TUG time, and SF-36 PF score were with the highest magnitude (*r* = 0.71, −0.73, and 0.56, resp.), indicating a large correlation. The total score showed moderate-to-small correlations with the hand grip strength and the Freiburger Physical Activity Questionnaire results (*r* = 0.45 and 0.27, resp.). The CS-PFP 10 subscores' correlations with the other measures were also in the predicted direction and magnitude; for example, the Timed Up and Go Test time correlated highly with the CS-PFP balance and coordination score (*r* = −0.73) and moderately with the CS-PFP upper body flexibility (*r* = −0.49).

#### 3.3.2. Internal Reliability

Internal consistency for the five subscores and total score of the CS-PFP 10 are provided in Table 5. Internal consistency (Cronbach's alpha) for the total score was *α* = 0.95. For the subscores, the coefficient ranged between *α* = 0.95 and 0.98, reaching high reliability for all subscores and the total score.

## 4. Discussion

In this study, we translated the CS-PFP 10 into German and set up the test laboratory in accordance with the guidelines with some approved (MEC) cultural adaptions. To our knowledge, this is the first time that a German version of the CS-PFP 10 has been validated.

Although physical function is a component of many functional performance assessments (e.g., Timed Up and Go Test [16], 6-minute walk test [27, 28], and Short Physical Performance Battery [4]), these tools often assess only a single task or a small aspect of functional performance and cannot sufficiently determine physical function in older adults who have beginning, subtle functional limitations. It is particularly advantageous to have one standardized, validated, reliable assessment that can be used in frail, vulnerable, and vigorous older adults. In everyday life, physical performance involves more than, for example, rising from a chair, turning around a pylon, and sitting down again, as tested in the TUG [16]. The CS-PFP 10 was developed to assess a broad range of physical functional performance of common, everyday tasks important to functional independence in older adults [7]. This test has been developed for English-speaking people living in the USA, yet there is a need for such functional, objective assessments that can be used in non-English-speaking countries, are well-translated, are adapted to the culture, and are validated to allow comparability of data [9].

Concurrent validity and internal consistency were investigated for the German version of the CS-PFP 10 in this study. The CS-PFP 10 is significantly related to concurrent measures of physical function (gait velocity, TUG, and hand grip strength) as well as self-reported measures (SF-36 physical functioning, Freiburger Physical Activity Questionnaire). The high correlations between the CS-PFP 10 total score and the gait velocity (*r* = 0.71) and with TUG times (*r* = −0.73) confirm the concurrent construct validity of the CS-PFP 10 for evaluating elements of walking ability and general mobility in older adults. The CS-PFP 10 endurance score showed the highest correlation with gait velocity (*r* = 0.72) and TUG times (*r* = 0.74), whereas the CS-PFP 10 upper body flexibility score showed the lowest correlation with these two measures of physical function (*r* = 0.55 and −0.49, resp.), as expected. The third performance-based measure, hand grip strength, showed low-to-moderate correlations with the CS-PFP 10 (values range between *r* = 0.22 and 0.47), except for the CS-PFP 10 upper body strength score (*r* = 0.60). This result seems plausible since the hand grip strength test measures a specific construct which targets most closely the construct of upper body strength. To our knowledge, there are no validity studies that compare the CS-PFP 10 with these measures of physical function. In relation to self-reported measures, the CS-PFP 10 total score highly correlated to the SF-36 physical functioning domain (*r* = 0.56). Cress et al. [7] reported a correlation coefficient of *r* = 0.75 between the CS-PFP 10 total score and the SF-36 physical function domain. The lower correlation coefficient in our study could be explained by two possible factors that affect the size of correlations. It is known from statistics that the value of *r* is affected by the amount of variability in the data [29]. In other words, the greater the range of scores in both variables, the greater the correlation between these variables. This factor is likely to be overlapped by the characteristics of the sample that can affect the size of *r*. In the present study, only community-dwellers were included; meanwhile, in the study of Cress et al. [7], community-dwellers as well as residents of a long-term care facility (including those who required assistance and those who did not) participated. Another possible reason for the lower value might be the fact that we used the CS-PFP short version of the test. Although there was a discrepancy in the amount of the correlation coefficient found, the high correlations support the similarity of the constructs.

Our study demonstrated excellent internal consistency of the translated version. Internal consistency reflects the extent to which items measure the same characteristics [30]. Cronbach's alpha coefficient of *α* = 0.95 of the total score found in this study was comparable with the result of the validation study of the original version [7]. The coefficients of the single subscores in this study ranged between *α* = 0.95 and 0.98 compared to the lower coefficients demonstrated in the original validation study (between *α* = 0.74 and 0.87). Discrepancies found can be explained by the differing test versions. The original validation study [7] was conducted with the 16-task CS-PFP test, whereas the present study was done with its short version of 10 tasks.

No floor or ceiling effects were observed in this study population. The CS-PFP 10 is targeted to assess abilities within a functional range and requires the participants to be ambulatory. In ambulatory individuals no floor effects have been seen. Since tasks are administered from easiest to most difficult, maximal information is gathered in a person who may not be able to complete all tasks. On the other hand, ceiling effects have not been observed even in the healthy adult populations showing that the CS-PFP 10 has the property of measuring functional performance among older adults with a wide range of physical ability. Due to this strength, the CS-PFP 10 is well suited to demonstrate change in physical function in exercise intervention studies [11, 31, 32] as well as in various disease conditions including Parkinson's disease [33], heart disease [34, 35], and fibromyalgia [36].

The CS-PFP 10 was recommended in a systematic review from 2012 [3] regarding geriatric screening and assessment as well as for scientific and research purposes in community-dwelling older adults. In contrast to the measurement of gait velocity, the CS-PFP 10 is more complex in use and requires significant space, which may restrict the fields of application. However, the clinical benefits of the CS-PFP 10 lie in the performance-based quantification of a broad spectrum of physical everyday functions which can provide, for example, general practitioners a more profound insight in the physical abilities of their older patients. Also, the tests subscores allow a more detailed localization of functional deficits that helps therapists target deficits from the beginning and quantify therapy success.

This study was conducted as part of a randomized placebo-controlled parallel group trial with healthy older adults. All participants understood and easily followed the test instructions. The time taken to complete the test was in average 50 minutes. However, only concurrent validity and internal reliability was tested in this substudy. Further study will be needed to test intrarater and interrater reliability as well as predictive validity of this assessment in German-speaking older adults.

## 5. Conclusion

The study reports the translation of the CS-PFP 10 into German and the site set-up in accordance with the CS-PFP 10 guidelines and the local cultural settings. Concurrent validity and internal reliability were established. In addition to the original version of the CS-PFP 10, this German version can be used to quantify physical functional performance in older adults who are primarily nondisabled and might exhibit ceiling effects for traditionally used functional performance measures.

## Figures and Tables

**Table 1:** Continuous-Scale Physical Functional Performance 10 Test—tasks and measurements.

	Measurements
*Low effort tasks*:	
(1) Kitchen pot carry	Time, weight
(2) Put on/take off jacket	Time
(3) Scarves pickup	Time
(4) Maximal reach	Distance
*Medium effort tasks*:	
(5) Floor sweep	Time
(6) Laundry loading/unloading	Time
(7) Sit down and get up from the floor	Time
*Hard effort tasks*:	
(8) Stair climbing	Time
(9) Grocery carrying and walking	Time, weight
(10) 6-minute walk	Distance

**Table 2:** Participant characteristics at baseline.

Variable	Total sample (*N* = 109)
Age (years)	74.1 ± 6.4
Gender	
Female	91 (83.5)
Male	18 (16.5)
MMSE score (points)	28.2 ± 1.6
Previous fall in the last 12 months	
None	34 (31.2)
1 or more	75 (68.8)
Fear of falling	
Yes	18 (16.5)
No	91 (83.5)
Height (cm)	165.9 ± 7.3
Weight (kg)	72.7 ± 15.4
BMI (kg/m^2^)	26.3 ± 4.7

*Notes*. Values are mean ± standard deviation or frequencies and percentage; MMSE = Mini Mental State Examination; BMI = Body Mass Index.

**Table 3:** Descriptive data of the measurements.

Variable	Minimum	Maximum	Mean	SD
CS-PFP 10 TOT	9.1	84.7	44.1	16.2
CS-PFP 10 UBS	12.4	97.8	46.4	18.7
CS-PFP 10 LBS	6.5	82.3	36.3	16.8
CS-PFP 10 UBF	18.7	83.4	56.0	13.0
CS-PFP 10 B&C	6.9	86.2	44.5	17.1
CS-PFP 10 END	9.1	87.5	45.8	16.8
Gait velocity	56.3	177.5	117.6	22.2
TUG	7.0	20.0	10.9	2.5
Hand grip strength	10.0	47.0	24.9	7.2
Freiburger PA	0.0	35.1	10.1	7.2
SF-36 PF	30.0	100.0	84.7	18.3

*Notes*. SD = standard deviation, CS-PFP 10 TOT = CS-PFP 10 total score, CS-PFP 10 UBS = CS-PFP 10 upper body strength score, CS-PFP 10 LBS = CS-PFP 10 lower body strength score, CS-PFP 10 UBF = CS-PFP 10 upper body flexibility score, CS-PFP 10 B&C = CS-PFP 10 balance and coordination score, CS-PFP 10 END = CS-PFP 10 endurance score, Freiburger PA = Freiburger Physical Activity Questionnaire, TUG = Timed Up and Go Test, and SF-36 PF = 36-item Short-Form General Health Survey, Physical Function subscore.

**Table 4:** Pearson's product moment correlation.

	Gait velocity	TUG	Hand grip strength	Freiburger PA	SF-36 PF
CS-PFP 10 TOT	0.71^*∗∗*^	−0.73^*∗∗*^	0.45^*∗∗*^	0.27^*∗∗*^	0.56^*∗∗*^
CS-PFP 10 UBS	0.60^*∗∗*^	−0.63^*∗∗*^	0.60^*∗∗*^	0.21^*∗∗*^	0.58^*∗∗*^
CS-PFP 10 LBS	0.69^*∗∗*^	−0.71^*∗∗*^	0.47^*∗∗*^	0.28^*∗∗*^	0.58^*∗∗*^
CS-PFP 10 UBF	0.55^*∗∗*^	−0.49^*∗∗*^	0.22^*∗*^	0.17^*∗∗*^	0.30^*∗∗*^
CS-PFP 10 B&C	0.70^*∗∗*^	−0.73^*∗∗*^	0.39^*∗∗*^	0.25^*∗∗*^	0.51^*∗∗*^
CS-PFP 10 END	0.72^*∗∗*^	−0.74^*∗∗*^	0.40^*∗∗*^	0.26^*∗∗*^	0.55^*∗∗*^

*Notes*. All correlations are statistically significant with ^*∗∗*^*p* < 0.01 or ^*∗*^*p* < 0.05; *n* = 109 except for SF-36 physical function, where only *n* = 105 were available; CS-PFP 10 TOT = CS-PFP 10 total score; CS-PFP 10 UBS = CS-PFP 10 upper body strength score; CS-PFP 10 LBS = CS-PFP 10 lower body strength score; CS-PFP 10 UBF = CS-PFP 10 upper body flexibility score; CS-PFP 10 B&C = CS-PFP 10 balance and coordination score; CS-PFP 10 END = CS-PFP 10 endurance score; Freiburger PA = Freiburger Physical Activity Questionnaire; TUG = Timed Up and Go Test; SF-36 PF = 36-item Short-Form General Health Survey, Physical Function subscore.

**Table 5:** Cronbach's alpha.

	*α*
CS-PFP 10 TOT	0.95
CS-PFP 10 UBS	0.97
CS-PFP 10 LBS	0.95
CS-PFP 10 UBF	0.98
CS-PFP 10 B&C	0.95
CS-PFP 10 END	0.95

CS-PFP 10 TOT = CS-PFP 10 total score, CS-PFP 10 UBS = CS-PFP 10 upper body strength score, CS-PFP 10 LBS = CS-PFP 10 lower body strength score, CS-PFP 10 UBF = CS-PFP 10 upper body flexibility score, CS-PFP 10 B&C = CS-PFP 10 balance and coordination score, and CS-PFP 10 END = CS-PFP 10 endurance score.
